# Respiratory function and bronchial responsiveness among industrial workers exposed to different classes of occupational agents: a study from Algeria

**DOI:** 10.1186/1745-6673-2-11

**Published:** 2007-10-08

**Authors:** Farid Ould-Kadi, Tim S Nawrot, Peter H Hoet, Benoit Nemery

**Affiliations:** 1 Faculty of Medicine, University of Oran, Oran, Algeria; 2 Occupational and Environmental Medicine, School of Public Health, KULeuven, Leuven, Belgium

## Abstract

Occupational exposures play a role in the onset of several chronic airway diseases. We investigated, in a cross-sectional study, lung function parameters and bronchial hyper-responsiveness to histamine in workers exposed to different airborne compounds.

The study group totalled 546 male subjects of whom 114 were exposed to welding fumes, 106 to solvents, 107 to mineral dust, 97 to organic dust and 123 without known exposure to airway irritants. A questionnaire was administered and spirometry and bronchial responsiveness to histamine were assessed by one observer, in the morning before work to prevent effects of acute exposure.

The mean (SD) age of the participants was 39.3 (7.8) years, with a mean duration of employment of 13.8 (6.6) years. Both before and after adjustment for smoking status, forced expiratory volume in 1 second (FEV_1_, expressed as % predicted) was lower in welders -4.0% (95% confidence interval [CI], -6.3 to -1.8; p = 0.01) and workers exposed to solvents -5.6% (CI: -7.9 to -3.3; p = 0.0009) than in control subjects. Furthermore, solvent workers had an odds ratio of 3.43 (95% CI: 1.09–11.6; p = 0.037) for bronchial hyperresponsiveness compared with the reference group.

The higher prevalence of bronchial hyperresponsiveness in solvent workers adds to the growing body of evidence of adverse respiratory effects of occupational solvent exposure. These results point to the necessity of preventive measures in solvent workers to avoid these adverse respiratory effects.

## Background

Although the dominant cause of chronic obstructive pulmonary disease (COPD) is cigarette smoking, there is little doubt that chronic occupational exposures to various agents contribute to the incidence and the severity of chronic airways disease, including COPD [1-4]. The quantitative contribution of occupational factors to the burden of COPD morbidity or mortality has been recently estimated at about 15% [5]. This value corresponds to the median of the attributable fractions of occupation to the occurrence of COPD, as derived from published population studies or occupational cohort studies.

These studies have been mainly concerned with occupational exposures to mineral dusts (in mines, metal industries or construction) or to organic dusts (in agriculture or agro-industry). The effects of exposure to irritant gases and vapors have not been investigated as much, and in particular the long-term respiratory effects of chronic occupational exposure to organic solvents are not well known [6].

Most epidemiological studies of the impact of occupation on the respiratory tract have used questionnaires and spirometry. Forced vital capacity (FVC) and Forced expiratory volume in one second (FEV_1_) are currently the best available functional measures and predictors of respiratory (and even general) health [7]. However, the individual risk factors that determine the susceptibility to an accelerated decrease in pulmonary function in smokers and/or occupationally exposed subjects are still largely unknown. One possibility is that nonspecific bronchial hyperresponsiveness is such a risk factor [8]. Although bronchial hyperresponsiveness has been assessed in many epidemiological studies, including in children (mainly in relation to asthma) [9], its prevalence and possible determinants have been studied in only few studies related to occupation [10-13].

In the present cross-sectional study, conducted in Algeria, pulmonary function and bronchial responsiveness to histamine were assessed in workers exposed to various common classes of agents, including mineral dusts, organic dusts, welding fumes and solvents. The main research question was whether the prevalence of bronchial hyperresponsiveness in these occupational groups differs from that in a control population of unexposed workers.

## Methods

### Study design

The survey took place between January and October 1996. Factories situated within a radius of 40 km of Oran, Algeria, and with presumed substantial exposure to one of the substances of interest (welding fumes, solvents, organic dust and mineral dust) and more than 20 workers employed, were selected. Eligible participants were men who had worked in the selected factories for at least two years. The control group included workers with life-long employment at the National Company for Gas and Electricity of Algeria (Sonelgaz) located in the same geographical area as the exposed workers. In total 620 workers fulfilling the selection criteria were selected, of whom 576 (93%) agreed to participate.

The group exposed to mineral dust comprised grinders from a metallurgical plant, quarry workers, underground mineworkers from a Kieselguhr (diatomite) mine, workers processing Kieselguhr, workers from a cement factory, and oven bricklayers from a steel factory. The group exposed to organic dust was composed of employees from five different cereal grain silos, working as loaders/unloaders or in cleaning/repairing jute bags to transport grain.

The group of welders came from a shipbuilding company and a metallurgic plant making water tanks; the metals welded (mainly steel) and the welding processes (mainly manual welding) were comparable in both plants. The group of solvent-exposed workers was composed mainly of workers from a paint manufacturing plant, and also spray-painters from the shipbuilding company. These subjects were exposed to xylene, toluene, white-spirit, ethyleneglycolacetate, methyl isobutyl ketone and butanol. The study was performed in accordance with the Helsinki Declaration and was approved by the ethical board of the University of Oran. We obtained informed written consent from the workers.

### Questionnaire

Data on smoking, respiratory symptoms, and diseases were collected by a face-to-face interview with questions based on the 1987 version of the European Coal and Steel Community respiratory questionnaire [14]. Non-smokers were defined as those who had never smoked regularly. Smokers were those who reported currently smoking at least one cigarette daily. Ex-smokers included those who had formerly smoked regularly. The questionnaire further gathered information on the following symptoms: chronic cough, chronic phlegm for as much as 3 months of the year; dyspnoea, defined as shortness of breath during low to moderate physical activity; symptoms suggesting asthma or allergy, the use of medication for asthma or allergy, and the presence of hay-fever and nasal allergies. Asthma was defined as answering "yes" to the question "Have you ever had asthma?". Allergic rhinitis was defined as answering "yes" to the question "Do you have hay-fever or any other kind of allergic rhinitis?"

### Clinical and functional measurements

The subjects were asked to refrain from smoking at least for one hour prior to testing. Spirometry and bronchial responsiveness were measured in the morning before work to prevent effects of acute exposure, by a single observer (F. Ould-Kadi). Height and weight were measured to the nearest cm and nearest 0.1 kg, respectively. FEV_1_, FVC and forced expiratory flows were obtained using an electronic spirometer (HI 298, ESSILOR) according to the ATS standards [15]. The ratio of FEV_1 _to FVC was calculated. Pulmonary function parameters were expressed as %-predicted according to Quanjer et al.[16,17]. After collection of the spirometric data, the same observer measured bronchial reactivity to histamine in subjects with a FEV_1 _of more than 60% predicted, according to the abbreviated protocol of Yan et al.[18] Histamine dichloride (Sigma, Belgium) was diluted in sterile 0.9% saline to concentrations of 10.2 μmol/ml (solution 1), 20.4 μmol/ml (solution 2), 81.5 μmol/ml (solution 3) and 163 μmol/ml (solution 4). Aerosols were generated using five DeVilbiss n°40 hand-operated glass nebulisers. In preliminary experiments, the average output of the five nebulisers was determined to be 0.03 g (range 0.028 to 0.039 g; SD: 0.008) for 10 actuations or 3 μl per actuation. Actuation of the aerosol was done at the start of an inhalation from functional residualcapacity to total lung capacity over 5 seconds, followed by a 3-second breath hold. The protocol involved one inhalation of saline (start value), then of solution 1 (0.03 μmol), then one inhalation of solution 1 (+0.03 μmol = 0.06 μmol cumulative), then three inhalations of solution 2 (+0.18 μmol = 0.24 μmol cumulative), then three inhalations of solution 3 (+0.73 μmol = 0.98 μmol cumulative), then 4 inhalations of solution 3 (+0.98 μmol = 1.96 μmol cumulative) and finally 4 inhalations of solution 4 (+1.96 μmol = 3.91 μmol cumulative). Sixty seconds after inhaling the aerosol, subjects performed three to five spirometry maneuvers (best quality effort selected) followed by inhalation of the next higher dose. Administration of increasing histamine concentrations was continued until FEV_1 _declined by 20% of baseline or the maximum cumulative dose was achieved (3.9 μmol). Subjects who had taken a beta-agonist within six hours of the examination were asked to withhold medication before returning for a later visit.

The histamine challenge test results can be expressed in a dichotomous way as the provocative dose of histamine causing a 20% fall in FEV_1 _(PD_20_) or in various other ways that take into account the entire dataset, even in those who do not reach a PD_20_. We calculated the area under the curve relating percent change in FEV_1 _against cumulative histamine dose, from control (0 μmol; starting FEV_1 _set at 100%) up to the highest dose tested (max 3.9 μmol).

### Statistical analysis

We used SAS software version 8.1 (SAS Institute Inc, Cary, NC) for statistical analysis. For comparison of means and proportions, we applied Student's t-test and the χ^2^-statistic, respectively. We used a general linear model and a logistic regression model to study group differences for continuous and dichotomous variables, respectively. Multiple regression models (lung function) and logistic regression models were adjusted for smoking, duration of employment, salary and reporting symptoms of allergy.

## Results

### Population characteristics

Of the 620 men, 576 (93%) agreed to participate, but 10 subjects were absent and 20 subjects with multiple exposures were excluded. Thus, the final study group totalled 546 subjects of whom 114 were exposed to welding fumes, 106 to solvents, 107 to mineral dust, and 97 to organic dust. The control group consisted of 123 workers without known significant exposures.

The characteristics of the 546 study participants are listed in Table 1. The mean (SD) age of the participants was 39.3 (7.8) years and was slightly but significantly higher in workers exposed to mineral and organic dust (Table 1). The mean duration of employment was 13.8 (6.6) years. Half the subjects (49%; n = 266) were current smokers, and 28% (n = 155) had never smoked. The mean cumulative history of smoking, among current smokers and past-smokers, was 13.3 (10.7) pack-years. The proportion of smokers was higher in welders (62%) and workers exposed to solvents (62%) compared with the controls (54%), while duration of employment and salary were significantly higher in the control group (Table 1). The reported symptom prevalences were generally very low, with only 112 subjects (20.5%) reporting at least one symptom (13.0% in controls, 18.4% in welders, 32.4% in solvent group, 21.5% in mineral dust group, 18.6% in organic dust group). Chronic cough was reported by 22 subjects (4.0%), chronic phlegm by 32 subjects (5.9%), wheezing by 50 subjects (9.2%), allergy by 35 subjects (6.4%) and asthma by 9 subjects (1.6%).

**Table 1:** Characteristics of the study population stratified by exposure group

	**Reference (n = 123)**	**Welders n = 114)**	**Solvents (n = 106)**	**Mineral dust (n = 107)**	**Organic dust (n = 97)**	**Total (n = 546)**	**overall p**
Age (years) Mean (SD)	38.3 ^1,2 ^(8.3)	37.6^ 1 ^(7.6)	39.3 ^1,2 ^(6.4)	40.1 ^2,3 ^(7.8)	41.8^ 3 ^(8.3)	39.3 (7.8)	0.0001
Height (cm) Mean (SD)	173 (6.7)	172.3 (5.9)	171.4 (6.7)	171.8 (6.8)	172.3 (5.8)	172.2 (6.4)	NS
Weight (kg) Mean (SD)	69.3 ^2,3 ^(10.9)	65.6^ 1 ^(10.5)	66.2 ^1,2 ^(11.5)	67.3 ^1,2 ^(11.6)	71^3 ^(12.4)	65.8 (11.5)	0.003
Duration exposure Mean (years) (SD)	18 ^4 ^(8.5)	13.9 ^2,3 ^(5.7)	11.9^ 1 ^(4.9)	12.8 ^1 ^(6.6)	14.6 ^3,4 ^(5.9)	13.8 (6.6)	<0.0001
Monthly salary (DA) Mean (SD)	11022 ^4 ^(1929)	8383 ^1 ^(1692)	9972^ 3 ^(1842)	9262 ^2 ^(2053)	9989 ^3 ^(1622)	9739 (2049)	<0.0001
Smoking Habit							
Non-smokers n (%)	43 (35)	27 (24)	26 (25)	29 (27)	30 (31)	155 (28.4)	NS
Ex-smokers n (%)	26 (25)	25 (22)	17 (13)	30 (28)	27 (28)	125 (22.8)	NS
Smokers n (%)	54 ^1 ^(44)	62 ^1,2 ^(54)	62 ^2 ^(59)	48 ^1 ^(45)	40 ^1 ^(41)	256 (48.8)	0.04
Cigarettes/day* Mean (SD)	15.2 ^1 ^(8.1)	17 ^1 ^(11)	18.9 ^2 ^(9.1)	16.4 ^1,2 ^(8.1)	17.3 ^1,2 ^(10.5)	16.9 (9.4)	NS
Pack years* Mean (SD)	12.9 ^1 ^(6.7)	12 ^1 ^(5.9)	16.5 ^2 ^(6.7)	11.9 ^1 ^(6.8)	13.1 ^1 ^(5.8)	13.3 (6.4)	0.04
Allergy n (%)	10 ^1,2 ^(8.1)	3 ^1 ^(2.6)	13 ^2 ^(12.4)	4 ^1 ^(3.8)	5 ^1,2 ^(5.1)	35 (6.4)	0.02
Asthma n (%)	3 (2.4)	3 (2.6)	2 (1.9)	0 (0)	1 (1)	9 (1.6)	NS

1,2,3: Groups with the same number in exponent do not differ significantly. *excluding never smokers.

DA: Algerian Dinar

Allergy based on reported symptoms, use of medication for allergy or the presence of hayfever or nasal allergies.

When compared to controls, only workers exposed to solvents had a significantly higher prevalence of symptoms, especially of chronic cough (8.6% vs 0.8%; *P *= 0.03) and chronic phlegm (12.4% vs 2.4%; *P *= 0.01). Smokers had a higher prevalence of at least one reported symptom (26.3%) than nonsmokers (14.2%) and exsmokers (16.0%), this being significant for chronic cough only (7.5% vs 0.6% and 0.8%, respectively).

### Baseline level of pulmonary function

Overall, FEV_1 _and FVC expressed as percent predicted,[16] were lower in smokers compared with non-smokers (97.6% *vs *102.1%; *P *< 0.0001 and 97.9% *vs *102.2%; *P *< 0.0001, respectively), and this was also true for the forced expiratory flows. The spirometric values of exsmokers did not differ from those of nonsmokers. Independently of smoking status, FEV_1 _tended to increase by 0.15% (SD: 0.08; *P *= 0.07) per year of employment.

Table 2 shows the pulmonary function variables according to the various classes of exposure. In general, the control group exhibited the highest mean values for all parameters and the group of solvent-exposed workers had the lowest values. In comparison with the control group, FVC and FEV_1 _were significantly lower in welders and workers exposed to solvents (Table 2). These differences remained significant, after adjustment by multiple regression for smoking status, years of employment and salary, with FEV_1 _being 4.0% (95% confidence interval [CI], -6.3 to -1.8; *P *= 0.01) lower in welders and 5.6% lower (CI: -7.9 to -3.3; *P *= 0.0009) in workers exposed to solvents. The other spirometry findings (FEV_1_/FVC, MEF_50, _MEF_75_) appeared not to be different across the different exposure groups (Table 2). The results were not altered when the adjustment for smoking was made by using number of pack-years instead of smoking status (not shown).

**Table 2:** Lung function stratified by exposure group

	**Reference (n = 123)**	**Welders (n = 114)**	**Solvents (n = 106)**	**Minerals dust (n = 107)**	**Organic dust (n = 97)**	**overall p**
FVC (%) Mean (SD)	103.9 ^3 ^(12.3)	99.5 ^1 ^(12.3)	97.8^ 1,2 ^(12.9)	101.5 ^2,3 ^(13)	102.5 ^2,3 ^(12)	0.03
FEV_1 _(%) Mean (SD)	102.7 ^3 ^(12.4)	98.3 ^1,2 ^(12.9)	96.2 ^1 ^(13.4)	101.1 ^2,3 ^(12)	101.8 ^2,3 ^(13.8)	0.01
FEV_1_/FVC (%) Mean (SD)	82.1 (6.0)	82.2 (6.2)	81.8 (7.3)	82.5 (5.8)	81.7 (6.8)	NS
PEF (%) Mean (SD)	92.5 ^3 ^(15.6)	87.1 ^2 ^(14.8)	81.8 ^1 ^(15.5)	88.5 ^2,3 ^(15.5)	90.6 ^2,3 ^(17)	<0.0001
MEF_25 _(%) Mean (SD)	89.7 ^2 ^(19.5)	84.3 ^1,2 ^(18)	80.8 ^1 ^(19.2)	86.6 ^2 ^(18.4)	88.4 ^2 ^(21.9)	<0.0001
MEF_50 _(%) Mean (SD)	85.9 (22.2)	81.5 (23.4)	79.3 (23.9)	82.9 (22.2)	85.1 (24.6)	NS
MEF_75 _(%) Mean (SD)	74.2 (21.6)	72.2 (20.8)	70.3 (24.6)	74.5 (20.1)	74.2 (23.1)	NS
MMEF (%) Mean (SD)	79 ^2 ^(21.2)	74.9 ^1,2 ^(22.8)	72 ^1 ^(22.7)	76.4 ^1,2 ^(19.5)	77.8 ^1,2 ^(23)	0.16
PD_20 _Number (%)	4 (3)^1^	6 (5)^1,2^	11 (11)^2^	6 (6)^1,2^	4 (4)^1,2^	NS

FVC (Forced Vital Capacity), FEV_1 _(Forced Expiratory Volume in 1 Second), PEF (Peak Expiratory Flow), MEF (Maximal Expiratory Flow at given percentage of FVC), MMEF (Maximal Mid-Expiratory Flow), all expressed as percent predicted (according to Quanjer et al. [15]), except for FEV_1_/FVC where real percentage is given (ratio × 100). PD_20_: number of subjects with a measurable PD_20 _(provocative dose of histamine leading to a 20% decrease in FEV_1 _with respect to the starting value) in the histamine test (n values of group lower by one in each group except in welders).

1,2,3: groups with the same number in exponent do not differ significantly

An obstructive impairment (FEV_1_/FVC < 0.70) was present in 24 subjects (4.3%, 13 smokers, 5 exsmokers), with 3 to 6 subjects only in each group (NS). A possible restrictive impairment (FVC and FEV_1 _< 80% predicted and FEV_1_/FVC > 0.70) was present in 11 subjects (2.0%, all smokers), with 1 subject in the control group, 4 subjects in the mineral dust group and 2 in each of the other three groups (NS).

### Bronchial responsiveness

The histamine test was not done in 4 subjects (one subject in each group, except welders) because of contra-indications. A decrease in FEV_1 _by 20% or more, i.e. a PD_20 _value, was obtained in 31 workers (5.7%) workers (Table 2); decreases in FEV_1 _by at least 15%, i.e. a PD_15 _value, or by at least 10%, i.e. a PD_10 _value, were obtained in 51 subjects (9.3%) and 95 subjects (17.4%), respectively. These prevalences were similar for nonsmokers, smokers or exsmokers.

The analysis of the histamine response using the Area Under the Curve (AUC) gave a mean value of 371 μmol.%FEV_1 _(range 312–412). Values higher than 390 were obtained in those whose FEV_1 _increased above the starting value. Among subjects without a detectable PD_20 _the mean value was 379 μmol.%FEV_1 _(range 320–412), and among subjects with a detectable PD_20 _the mean value was 251 μmol.%FEV1 (range 312–346). Neither for the dichotomous (PD_20_) nor the continuous (AUC) variables of bronchial hyperresponsiveness, was there a relation with age, smoking, the duration of employment, or symptoms of allergy. There was also no interaction between age and smoking for these parameters. However, the odds of having a detectable PD_20 _was 18.8 (95% C.I. 4.5–79.1, *P *< 0.001) in those reporting asthma symptoms (9 subjects).

**Figure 1:**
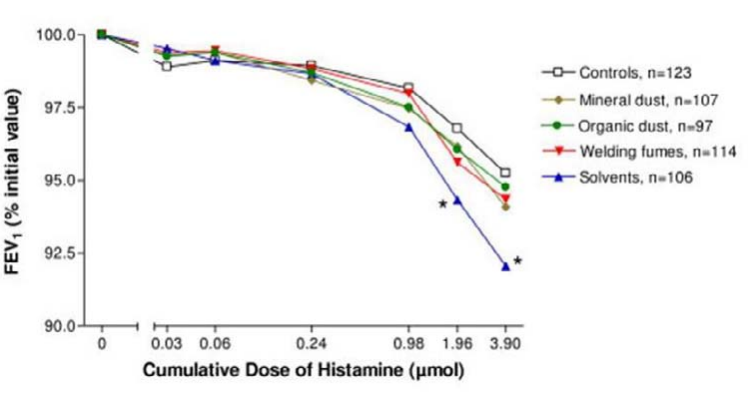
**Histamine responsiveness**. Mean FEV_1 _as the percentage of the initial value (0) after increasing doses of inhaled histamine, administered by aerosol by a hand held nebuliser, according to exposure group. Error bars have been deleted for clarity. * denotes significant difference (p < 0.05) compared with controls. At the higher doses the numbers of subjects are slightly lower than indicated in the legend because the test was interrupted when FEV_1 _decreased by 20% or more (i.e. detectable PD_20_, see table 2 for the number of subjects with a detectable PD_20 _in each group).

The presence of bronchial hyperresponsiveness, defined as a measurable PD_20_, was more frequent in solvent workers compared with controls (11% *vs *3%; *P *= 0.028), yielding an odds ratio for bronchial responsiveness of 3.43 (95% CI: 1.05–11.1; *P *= 0.04) in solvent workers compared with controls, independently of the aforementioned covariates. Using the area under the curve as a continuous measure of bronchial responsiveness, confirmed the dichotomous analysis, before (figure 1) and after adjustment for the same covariates: the AUC was 2.9% (CI: -0.9% to -4.7%; *P *= 0.04) lower in workers exposed to solvents compared with the controls. However, no significant differences were obtained for the other groups.

## Discussion

Key findings in our study are that workers exposed to metal fumes and solvents had a lower baseline lung function and that solvent-exposed workers had a 3.4 times higher risk of having nonspecific bronchial hyperresponsiveness than the reference group.

### Respiratory symptoms

In this population the prevalence of respiratory symptoms was low. Apart from the fact that this was a relatively young working population, it is possible that the respondents were fearful of admitting symptoms and/or that the questionnaire utilized [14] did not capture respiratory symptoms as well as in the European populations where it was developed. Nevertheless, as expected, smokers reported more symptoms than nonsmokers and exsmokers.

The prevalence of asthma (1.6%) and allergy (6.4%) also appeared to be very low. Again, this may reflect a healthy worker effect or be due to a validity issue of the questionnaire utilized, but it is also compatible with the low prevalence of atopy and asthma in North Africa, at least in children [17].

### Pulmonary function

The spirometric data were generally well within the range of normality as defined by the prediction equations of Quanjer et al.[16]. Smokers had slightly but significantly poorer values than nonsmokers and exsmokers, which indicates that the quality of the measurements was adequate. The trend (*P *= 0.07) for an improvement in FEV_1 _with duration of employment may be due to a healthy worker effect.

Only few data on pulmonary function have been published from populations with occupational exposure to solvents. A cross-sectional study on the association between pulmonary function and solvent exposure in workers of an automobile paint and coating plant showed a negative correlation between FEV_1 _and years of solvent exposure [19]. Data on 15,637 people aged 20–44, randomly selected from the general population of 26 areas in 12 industrialised countries showed that the highest risk of asthma, defined as bronchial hyperresponsiveness and reported asthma symptoms or medication, was observed for farmers (odds ratio 2.62 [95% CI 1.29–5.35]), painters (2.34 [1.04–5.28]), plastic workers (2.20 [0.59–8.29]), cleaners (1.97 [1.33–2.92]), and spray painters (1.96 [0.72–5.34])[20]. In a cross-sectional study in a sample of furniture workers exposed to isocyanate paints, the risk of asthma in the exposed group was 2.1% versus 0.8% in controls (*P *= 0.07)[21]. There was no recorded evidence for the use of polyurethane paints in the present group.

The group of welders also had a slightly poorer pulmonary function. Our findings are consistent with those from Akbar-Khanzadeh [22] who reported a greater deterioration of lung function with advancing age in welders compared with controls. In a longitudinal study of welders and caulker-burners with follow-up of retired workers, Chinn and colleagues [23] demonstrated that FVC, FEV_1_, PEF, and FEF_50% _declined over time; the decrease was caused equally by welding and smoking. In 286 students entering an apprenticeship programme in the welding profession FEV_1 _dropped on average by 8.4% (P = 0.01) during the follow-up of 15 months [23]. However, in contrast to the above results, several investigators have found no overall effect of welding on lung function. Our study included welders in confined and poorly ventilated spaces, like shipbuilding. The contradictory results regarding lung function in welders could be caused by differences with regard to healthy worker selection, smoking habits, co-exposure to asbestos, workplace variability, the welding materials used, the amount of ventilation, and the kinds of protective measures taken.

The functional impairment observed in solvent-exposed workers and welders was not entirely typical for bronchial obstruction since FEV_1 _and FVC were decreased to a similar extent. In the absence of measurements of total lung capacity, it is not possible to attribute the observed changes to lung restriction. The number of subjects with FEV_1 _and FVC values below 80% pred. with FEV_1_/FVC > 0.70 (2 in each category) was low and it did not differ significantly from the numbers observed in the controls. It is possible that exposure to some occupational agents, and solvents in particular, reduces both FEV_1 _and FVC, as shown, for instance, in recent studies of workers exposed to coke oven emissions [24], cement dust [25] or dust from the collapsed World Trade Center [26].

In contrast to some other reports [24,27,28], we did not observe adverse respiratory effects of exposures to organic dust and mineral dust. Individuals susceptible to adverse respiratory effects from organic or mineral dust may have quit work and therefore dropped out of the exposed group. This may explain the higher mean FVC among workers exposed to mineral dust. In the current study, FVC and FEV_1 _increased marginally with years of employment suggesting that a healthy worker effect might have occurred and weakened the observed associations. Because of the cross-sectional nature of this study, it is not possible to differentiate the effects of current exposure from those of cumulative exposure. Another limitation is that we had no exposure measurement data, neither at the individual nor at the group level.

### Nonspecific bronchial hyperresponsiveness

In the present study, bronchial responsiveness to histamine was not influenced by smoking status. Smoking per se does not appear to affect airway responsiveness. Although as a group smokers have somewhat higher bronchial responsiveness than nonsmokers, this difference disappears when baseline airway calibre (FEV_1_) is taken into account [29]. Also, smoking and atopy act synergistically to increase airway reactivity [30], but this was not apparent in the present population, probably because there were only few atopic subjects.

We studied bronchial hyperresponsiveness using histamine as the bronchoconstrictor, as in the abbreviated protocol of Yan et al. [18]. Even though histamine and methacholine are not fully interchangeable, both agents provide concordant results [31]. We studied bronchial responsiveness both as a dichotomous variable (PD_20_) and as a continuous variable. A detectable PD_20 _is used clinically, because it is simple to understand and it is clinically relevant. However, such dichotomous response only gives useful information for those subjects having a measurable PD_20_. Replacing a parameter that is continuous with one that is dichotomous is not only arbitrary but results also in less phenotypic precision, especially for epidemiological studies. Therefore, continuous measures of bronchial hyper-responsiveness have been proposed, such as that of O'Connor et al. [30] or the BRindex [32]. A disadvantage of the latter two methods is that they discard information as well, since they assess the percentage fall in FEV_1 _at the highest dose relative to baseline. Hence, these two measures need to be used with caution because they are largely influenced by "error" in the fall of FEV_1 _at the final dose. This is why we chose to calculate the area under the curve relating the % change in FEV_1_against cumulative histamine dose from 0 to 3.9 μmol. To our knowledge, this has not been done by others.

As indicated in the introduction, only few data are available concerning bronchial responsiveness in adult working populations. In a cross-sectional study of 688 male workers, Kremer et al. [13] found no association between low grade exposure to various airway irritants and airway hyperresponsiveness, which was determined both as PC_20 _and as a slope according to O'Connor [30]. That study did not contain solvent-exposed painters or welders. Beckett et al. [10] measured spirometry and methacholine reactivity annually for three years in 51 welders and 54 non-welder control subjects: no effect of welding was found on methacholine reactivity, neither at baseline, nor during follow-up. This confirmed negative findings from a smaller study of welders [33]. In the European Community Respiratory Health Survey (ECRHS) associations were studied, in 13,253 men and women of 20 to 44 y, between occupational exposures and various indices, including spirometry and methacholine responsiveness [11]. Although some occupational exposures (especially agriculture) were found to contribute to bronchitis symptoms, neither lung function, nor bronchial responsiveness were related to any of the occupational exposures indices, none of which, however, included solvents as a specific category [11].

On the basis of both PD_20 _and the AUC method for expressing bronchial responsiveness, we found that solvent exposed workers had a higher bronchial response to histamine. However, with the present data it cannot be determined whether the higher bronchial responsiveness reflects the somewhat lower FEV_1 _in this group or whether they had a lower FEV_1 _because they had bronchial hyperresponsiveness. In the latter case, this would strengthen the hypothesis that bronchial responsiveness is a risk factor for an accelerated decline in ventilatory function [8].

Research on occupational safety and health is occasionally carried out jointly between the industrialized and developing countries. The present study must be interpreted within the context of its limitations. Observational studies cannot prove causation. Occupational health remains limited in Northern Africa because of competing social, economic, and political challenges. Although no quantified exposure data were available, it might be assumed that compared with North-American and West-European standards, high exposure to the studied agents occurred since no or very little preventive measures were adopted in these Algerian work places at the time of the study. Besides limited or no quantified exposure and the rather low duration of employment, other factors might have biased our estimates. Thus, although the control group also consisted of blue-collar workers, these proved to have a higher income and to smoke less. This difference in socioeconomic status may be unfortunate for the purposes of the study, but such confounding should not be too surprising: healthier jobs are often paid better and this can be expected to lead to better nutrition and lifestyle [34].

In conclusion, baseline FEV_1 _was lower in smokers and, independently of smoking status, lower in workers exposed to solvents and metal fumes. Further, our results showed an increased prevalence and degree of bronchial hyperresponsiveness in solvent workers compared with controls.

## Competing interests

The author(s) declare that they have no competing interests.

## Authors' contributions

All authors took part in the interpretation of the results and prepared the final version. FOK and BN designed the study. FOK recruited the subjects, administered the questionnaires, performed spirometry and bronchial reactivity to histamine and constructed the database. TN and PH did the statistical analysis.
